# Characterization of Mitochondrial Double-Stranded RNA Levels in Non–Small Cell Lung Carcinoma

**DOI:** 10.1158/2767-9764.CRC-25-0656

**Published:** 2026-04-07

**Authors:** Matthew R. Krieger, Sandy Che-Eun S. Lee, Ting Fu, Maya Cielo Cornejo, Kevin L. He, Ryan Howe, Vanessa Saldivar, Angela L. Zhang, Guillaume F. Chanfreau, Michael A. Teitell, Xinshu Xiao, David B. Shackelford, Carla M. Koehler

**Affiliations:** 1 Department of Chemistry and Biochemistry, University of California, Los Angeles, Los Angeles, California.; 2 Pulmonary, Critical Care Medicine, David Geffen School of Medicine, University of California, Los Angeles, Los Angeles, California.; 3 Jonsson Comprehensive Cancer Center, UCLA, Los Angeles, California.; 4 Department of Integrative Biology and Physiology, University of California, Los Angeles, Los Angeles, California.; 5 Molecular Biology Institute, University of California, Los Angeles, Los Angeles, California.; 6 Department of Pathology and Laboratory Medicine, David Geffen School of Medicine, University of California, Los Angeles, Los Angeles, California.

## Abstract

Mitochondrial double-stranded RNA (mtdsRNA) is exported to the cytoplasm when mitochondrial RNA degradation is impaired, serving as a novel damage-associated molecular pattern for mitochondrial stress. Although mtdsRNA has been detected in certain cancers, its prevalence and functional role remain largely underexplored. Moreover, mtdsRNA is not readily detectable in large-scale computational datasets, reflecting technical limitations in the detection of structured mitochondrial transcripts. In this study, we used comprehensive computational characterization of non–small cell lung cancer (NSCLC) cell lines and identified elevated light-strand transcripts in a subset of cell lines, suggesting a potential for mtdsRNA formation. We stratify NSCLC lines into groups with high and low mtdsRNA abundance. Despite high cytoplasmic mtdsRNA levels in select NSCLC cell lines, we did not identify significant correlation with mtdsRNA abundance and type-I interferon (IFN-l) response. RT-qPCR analysis revealed that only USP18 transcripts amongst the IFN-l transcripts probed were significantly regulated in select NSCLC lines, indicating a partial or suppressed IFN-I response. Strand-specific RT-qPCR also revealed no bias in mitochondrial gene expression. These findings indicate that basal mtdsRNA accumulation alone is insufficient to trigger IFN-I signaling and may be tolerated in NSCLC, indicating adaptive mechanisms. Our findings suggest that mtdsRNAs could serve alternative nonimmunogenic roles in tumor biology. Importantly, our work reports that mtdsRNA is upregulated in a subset of NSCLC cell lines and in other cancer cell types, suggesting that mtdsRNA may serve as a new marker of mitochondrial dysfunction in cancer.

**Significance:**

Our study identified and characterized NSCLC cell lines with elevated mtdsRNAs, suggesting that mtdsRNA may be a new signaling molecule in lung cancer for mitochondrial stress. We identified that accumulation of mtdsRNAs did not necessarily activate IFN-l response, indicating that NSCLC cell lines may have adaptive mechanisms to reduce IFN-l response.

## Introduction

**Figure 1.**
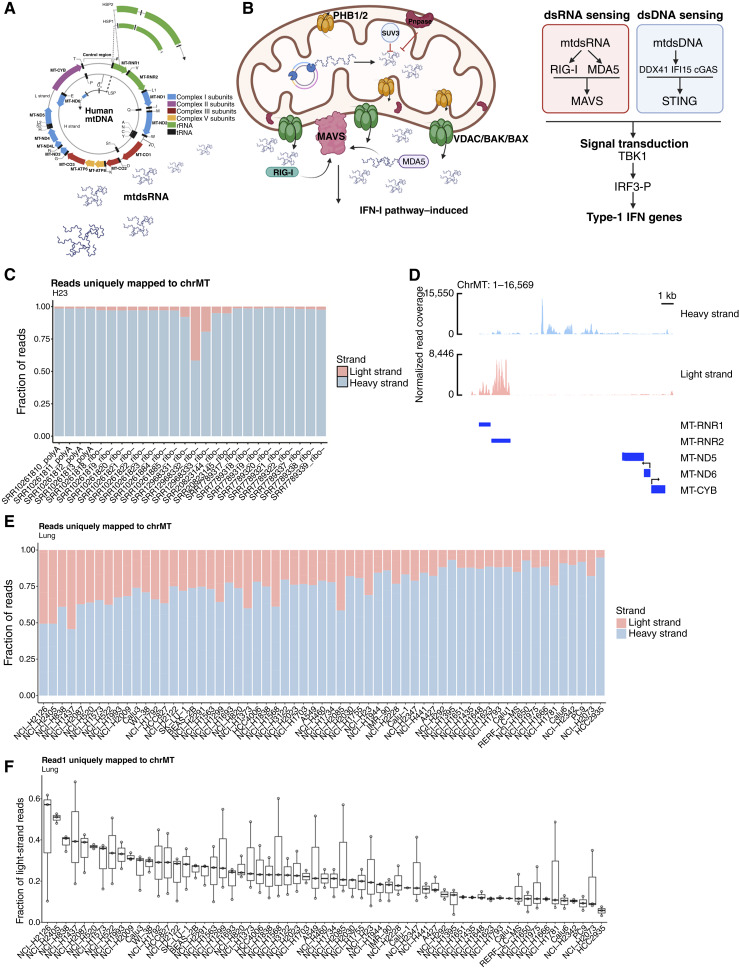
mtdsRNA is detected across NSCLC cell lines. **A,** Schematic of the mitochondrial genome and transcription promoters. **B,** Schematic of dsRNA export in mitochondria (left) and sensing pathways for dsRNA and dsDNA in cells (right). **C,** Fraction of reads mapped to light (blue) and heavy (red) strands of mitochondrial genome (ChrMT) detected in H23 (*n* = 3) from various RNA-seq data sets. **D,** Per-base normalized read coverage of reads mapped to the heavy (blue) and light (red) strands of the mitochondrial genome in H23. **E,** Fraction of reads mapped to light (blue) and heavy (red) strands of mitochondrial genome (ChrMT) detected in each cell line (*n* = 3). **F,** Box plot of fraction of reads mapped to the light strand of chrMT in each cell line. Box plot represents three replicates. [**A,** Created in BioRender. Lee, S. (2026) https://BioRender.com/xnm6tcy].

Mitochondrial DNA (mtDNA) is transcribed bidirectionally, leading to the synthesis of both heavy (H)- and light (L)-strand encoded RNAs (Fig. 1A; refs. 1, 2). Under physiologic conditions, rapid degradation of the L-strand transcript prevents the formation of the mitochondrial double-stranded RNA (mtdsRNA; refs. 1–3). Disruption of this process, such as through loss and/or mutation of polyribonucleotide nucleotide transferase (PNPT1), results in accumulation and release of mtdsRNA into the cytoplasm (3–5). Cytoplasmic mtdsRNA subsequently activates the type-I interferon (IFN-l) signaling pathway (3, 5), suggesting a feedback mechanism that mitigates mtdsRNA-induced cellular stress. These findings collectively support the role of mtdsRNA as a novel damage-associated molecular pattern (DAMP; Fig. 1B; refs. 3, 5, 6). Although the accumulation of mtdsRNA and its associated viral mimicry effects have been described in various cellular contexts (7–11), their role in tumor biology and mitochondrial dysfunction remains understudied.

Despite the conceptual appeal of mtdsRNA as a readout of mitochondrial stress and innate immune activation, several technical and biological challenges complicate its development as a reliable biomarker. mtdsRNAs are heterogeneous in length and structure, present at low abundance, and highly susceptible to degradation, limiting sensitive and specific detection across platforms (3, 5). Moreover, distinguishing mitochondrial-derived dsRNA from nuclear or retroelement-derived dsRNA remains challenging in bulk sequencing and antibody-based approaches that lack organelle-level resolution (12).

Biologically, mtdsRNA abundance is tightly regulated by mitochondrial transcription, RNA surveillance pathways, and innate immune sensing mechanisms (13), all of which may be rewired or adapted across different cancer types. In immune-cold tumors, attenuated antiviral signaling may further decouple mtdsRNA accumulation from canonical downstream IFN responses (14, 15), complicating interpretation. These considerations highlight the need for integrative strategies that pair molecular specificity with functional context upon evaluating mtdsRNA as biomarkers.

Notably, at least 45% of patients with non–small cell lung cancer (NSCLC) harbor KRAS/LKB1 mutant tumors, which are typically immune-cold and resistant to conventional therapies (16–18). These patients are in urgent need of novel biomarkers that can stratify tumors beyond their immunogenic potential. The presence of mtdsRNAs may serve as a biomarker of mitochondrial stress, particularly in immune-cold tumors in which antiviral signaling is dampened. Furthermore, the accumulation of mtdsRNAs could inform therapeutic strategies that exploit metabolic vulnerabilities. Here, we comprehensively characterize the abundance and functional relevance of mtdsRNAs across NSCLC cell lines and define their canonical role in antiviral signaling in immune-cold contexts. Through integrative computational analysis and experimental validation, we demonstrate that mtdsRNAs can serve as a complementary proxy for tumor subtype detection and patient stratification. Our work provides a valuable resource in understanding the biological importance of mtdsRNA in NSCLC cell lines.

## Materials and Methods

### Reagents

All reagents used in the study can be found in Supplementary Table S1.

### Images

Figures have been created using BioRender.

### mtdsRNA analysis

Stranded RNA sequencing (RNA-seq) datasets for the H23 lung cancer cell line was obtained from the NIH Sequence Read Archive (SRA) data portal under the following accessions: SRP225193, SRP335873, SRP159657, SRP431131, SRP156785, SRP290873, SRP159660, and SRP389901. RNA-seq data for an additional 60 lung cancer cell lines were also retrieved from the NIH SRA data portal (accession: SRP290873; ref. 19).Stranded RNA-seq datasets for the prostate cell lines were obtained from the NIH SRA data portal under the following accessions: PRJNA997353, PRJEB50063, PRJNA835419, PRJNA844003, PRJNA321055, and PRJNA759043. Patient RNA-seq data for lung adenocarcinoma (LUAD), lung squamous carcinoma (LUSC), colon adenocarcinoma (COAD), and breast cancer generated by the Clinical Proteomic Tumor Analysis Consortium (CPTAC) were obtained from the Genomic Data Commons portal (https://portal.gdc.cancer.gov/). Initial quality assessment of raw sequencing reads was performed using FastQC (v0.11.8). Adapter sequences and low-quality bases (Phred score <20) were trimmed using BBDuk (v35.85) with the following parameters: k-mer size = 25, minimum k-mer length = 11, and a Hamming distance of 1. Quality trimming was applied to both ends of the reads, and any reads shorter than 35 base pairs after trimming were discarded. FastQC was rerun on the processed data to confirm the removal of low-quality reads and adapter contamination. Trimmed reads were aligned to the human reference genome using HISAT2 (v2.2.1; ref. 20) to reduce false positives from multi-mapping between mitochondrial and nuclear genomes. Soft clipping (–no-softclip) and discordant alignment (–no-discordant) options were disabled, as the reads had already been trimmed and were expected to align concordantly as paired-end reads. Only uniquely mapped reads were retained for downstream analysis.

Given the stranded nature of the libraries, a custom Python script was used to verify and record the strand orientation of the reads. For each sample, the total number of reads mapping to the mitochondrial chromosome (chrMT) was extracted from the SAM files using SAMtools (21) with flags applied to filter based on read directionality, strandedness, and proper pairing. The proportions of reads mapping to the forward (light) and reverse (heavy) strands of chrMT were calculated and visualized in R (R Core Team, 2024). The mitochondrial read distribution was visualized using SparK (bioRxiv 2025.12.07.692871).

### dsRNA immunoprecipitation and sequencing

#### dsRNA immunoprecipitation

Cells were seeded to 15-cm plates and lysed in NP-40 lysis buffer (Invitrogen) for 30 minutes on ice. Protein A/G beads (Invitrogen) were incubated with J2 antibody overnight at 4°C. Conjugated beads with J2 antibody were then incubated with the lysates for 2 hours at 4°C. The beads were then washed with high-salt phosphate-buffered saline (PBS) buffer (1x PBS, 0.1% Tween, 1 mol/L NaCl) once, followed by two washes with a low-salt PBS buffer (1x PBS, 0.1% Tween, and 0.5 mol/L NaCl). The immunoprecipitated samples were eluted from the beads using 1 mg/mL proteinase K (Thermo Fisher Scientific) in NP-40 lysis buffer.

#### Nanopore sequencing

PCR-cDNA libraries were prepared from ∼100 ng of *in vitro* polyadenylated dsRNAs using the PCR-cDNA sequencing kit from Oxford Nanopore (Oxford Nanopore Technologies, cat. #SQK-PCB114.24) and pooled as per the manufacturer’s instructions. Sequencing was performed using R10.4.1 flow cells on a MinION Mk1B device and sequenced for 24 hours. Basecalling was performed using Dorado (version 7.9.8).

### Sequencing alignment, quantification, and normalization

Reads for cDNA-PCR sequencing: Reads were base-called in real-time using the default algorithm on MinKNOW (v.6.8.11) with Dorado (v.7.11.2). Reads were then mapped to the mitochondrial genome using Minimap 2 (v.2.17-r941) using the parameter “minimap2-a.” Visualization of the reads was done using IGV (v.2.12.3). Alignments were sorted and indexed using Samtools v1.10. BED files were generated from the BAM files using bedtools bamtobed.

Histograms were generated by separating reads in the BED files by strand and normalizing the coverage to the genomic maximum for that sample. Bar graphs were generated by counting all the reads that unambiguously aligned using the FeatureCounts function in the Rsubread package with the following parameters: isGTFAnnotationFile = TRUE, GTF.featureType = c(“gene”), GTF.attrType = “Name”, isLongRead = TRUE, countMultiMappingReads = FALSE, allowMultiOverlap = TRUE, minOverlap = 6, useMetaFeatures = FALSE, and nthreads = 8. Percentages from FeatureCounts for all samples were then used in the creation of bar graphs.

### Long read sequencing

Cells were harvested from 10-cm plates, and genomic DNA was extracted using the EasyPrep Genomic DNA Mini Kit (Bioland Scientific LLC). DNA concentration and purity were measured with a Thermo Scientific NanoDrop UV-Vis spectrophotometer. PCR amplifications were performed using 1,000 ng or 2,000 ng of genomic DNA as template. Three overlapping regions spanning the mitochondrial genome were amplified: RNR2 to COX3 (7,219 bp), COX3 to ND5 (4,573 bp), and ND5 to RNR2 (5,160 bp). Each 50 μL PCR reaction contained 1X Taq buffer (NEB), 200 μmol/L of each dNTP (NEB), 0.2 μmol/L forward primer, 0.2 μmol/L reverse primer, nuclease-free water (Invitrogen), and Taq DNA polymerase (NEB). PCR amplification was done on a Biometra TOne thermocycler (Analytik Jena) with the following cycling conditions: initial denaturation at 95°C for 2 minutes; 30 cycles of 95°C for 30 seconds, 55°C for 30 seconds, and 68°C for 7 minutes, followed by a final extension at 68°C for 10 minutes. PCR products were analyzed by electrophoresis on a 1% agarose gel stained with ethidium bromide (Company, catalog number) and visualized under UV illumination (Bio-Rad). PCR products demonstrating correct band size were then sent off for sequencing (Plasmidosaurus).

### Long read sequencing analysis

Three overlapping PCR-amplified regions were sequenced using a commercial sequencing service. The resulting sequence fragments were assembled using SnapGene software (v6.2.2) to reconstruct the full plasmid sequence. The reconstructed plasmid was oriented to match the reference genome sequence on a gene-by-gene basis. Subsequently, base pairs were aligned with corresponding RNA-seq reads according to gene locations. Guanine (G) nucleotides with lower read percentages were identified, and their base composition and frequencies were cataloged across all three RNA-seq replicates. DNA-seq data from three replicates were then compiled, and the identified G sites were compared at the DNA level. Sites in which all three DNA replicates contained adenine (A) at the corresponding position were designated as potential adenosine deaminase acting on RNA (ADAR)-editing sites.

### RNA read count analysis

RNA-seq .bam files were processed using SAMtools to obtain mapped, unmapped, and total read counts. Mitochondrial reads were quantified using FeatureCounts, excluding duplicate and overlapping reads. Aligned reads were counted using either the complete human mitochondrial genome annotation or a modified annotation file in which all tRNA-related genes and exons were removed.

### Cell culture

Cells were obtained from either the ATCC or the Shackelford Lab. All cells were cultured in high-glucose Dulbecco’s modified Eagle medium (Invitrogen). All media were supplemented with 10% fetal bovine serum (Gibco), 100 U/mL penicillin, and 100 μg/mL streptomycin (1%). Cells were cultured under 5% CO_2_ in air at 37°C. All cell lines were tested for short tandem repeats to verify cell line of use and tested for *Mycoplasma* using a Mycoalert kit (Lonza).

### Immunofluorescence

Cells were seeded in six-well slides with × mm glass cover slips and fixed in 4% paraformaldehyde for 15 minutes at room temperature. Cells were permeabilized using 0.25% Triton X-100 in PBS for 15 minutes. Samples were blocked with 5% bovine serum albumin in PBS for 1 hour. Appropriate primary antibody was applied for 1 hour. Coverslips were washed three times. Secondary antibody was applied for 1 hour. Coverslips were washed three times. All washing was performed with 0.1% Tween 20 in PBS. Coverslips were mounted with Antifade Mounting Medium (Thermo Fisher Scientific) before imaging. Fluorescence imaging was done using 3i Spinning Disk Confocal microscope (Marianas) consisting of CSU-X1 A1 Spinning Disk (Yokogawa) attached to a Zeiss Axio Observer 7 microscope (Zeiss). Standard filter sets were used for imaging. The Pearson’s correlation coefficient (PCC) was calculating using JACoP plugin on ImageJ.

### Immunoblotting

Cells were washed twice with PBS and lysed in radioimmunoprecipitation assay buffer (containing 50 mmol/L Tris–HCl, pH 7.5, 150 mmol/L NaCl, 1% NP-40, 0.1% SDS, 0.5% sodium deoxycholate, 1 mmol/L EDTA, and 1 mmol/L phenylmethylsulfonyl fluoride) for 30 minutes on ice. Samples were then centrifuged at 21,000 *g* for 10 minutes at 4°C. The supernatant was collected and measured for its total protein concentration by bicinchoninic protein assay (Thermo Fisher Scientific) using a x plate reader. Samples were then prepared with 5x Sample Buffer (125 mmol/L Tris base, 5 mmol/L EDTA, 1 mol/L glycine, and 10 mmol/L SDS) and β-mercaptoethanol (EMD Millipore) and boiled at 95°C for 5 minutes. Proteins were resolved on SDS-polyacrylamide gel electrophoresis gels and transferred to a polyvinylidene difluoride membrane (Invitrogen) and blocked in 5% milk. Membranes were incubated with primary antibodies overnight at 4°C and secondary antibodies for an hour. All antibodies used can be found in Supplementary Table S1.

### RNA extraction and qPCR

Total RNA was extracted from cells using GeneJet RNA Purification Kit (Invitrogen) and collected into tubes as per the manufacturer’s protocols. RNA concentration was determined using NanoDrop (Thermo Fisher Scientific). RNA with a minimum of A260/A280 ratio of 1.8 was used. Reverse transcription was performed using qScript cDNA SuperMix (Bio-Rad) according to the manufacturer’s instructions. PCR analysis was performed using SYBR Green Supermix (Roche). The cycling parameters were as follows: 95°C for 30 seconds, 95°C for 15 seconds for 39 cycles, 60°C for 50 seconds for 39 cycles, and 65°C for 5 seconds. Primer sequences can be found in Supplementary Table S1.

### Statistical analysis

Unless otherwise specified, data were analyzed with the use of GraphPad Prism 6 (GraphPad Software Inc.) with either unpaired Student *t* test or one-way analysis of variance with Bonferroni correction.

## Results

### NSCLC cell lines reveal enrichment of dsRNA

We analyzed publicly available double-stranded RNA-seq datasets from the H23 lung cancer cell line in order to quantify the fraction of reads mapped to the mitochondrial genome, distinguishing between the light and heavy strands, based on a previous study in which we demonstrated that the H23 lung cancer cell line has high levels of mtdsRNAs (5). Using dataset SRR12968332 (19), H23 exhibited a high proportion of reads mapping to the light strand (Fig. 1C), suggesting the possible presence of mtdsRNAs formed between the light and heavy strands. Other RNA-seq datasets for H23 lacked reads that mapped to the light strand. This was not surprising because mtRNAs are often underrepresented in datasets, because mitochondrial (mt) RNA lacks typical PolyA tails and insufficient data processing pipelines discard reads that map to the mitochondrial genome (22). To investigate this further, we examined the specific mapping locations of the light-strand reads in SRR12968332. We found that most of these reads aligned to the light strand within or antisense to the mitochondrial genes *RNR1* and *RNR2* (Fig. 1D). We then extended our analysis to 60 additional lung cancer cell lines with similarly prepared stranded RNA-seq libraries (19). Strikingly, many of these cell lines also showed a substantial fraction of reads mapping to the mitochondrial light strand. H2126 and H2405 cells displayed light-strand mapping fractions as high as 50% of total mitochondrial reads (Fig. 1E and F). To define quantitative groups of mtdsRNA abundance (high, medium, and low), we classified the fraction of light reads detected across cell lines into quartiles (Supplementary File S1). Cell lines in the top and bottom quartiles were designated as having high and low mtdsRNA levels, respectively, whereas those falling between these quartiles were classified as intermediate. Together, these analyses reveal that a subset of NSCLC cell lines exhibits pronounced mitochondrial light-strand transcripts.

### mtdsRNA in NSCLC reveals minimal ADAR-editing sites

**Figure 2.**
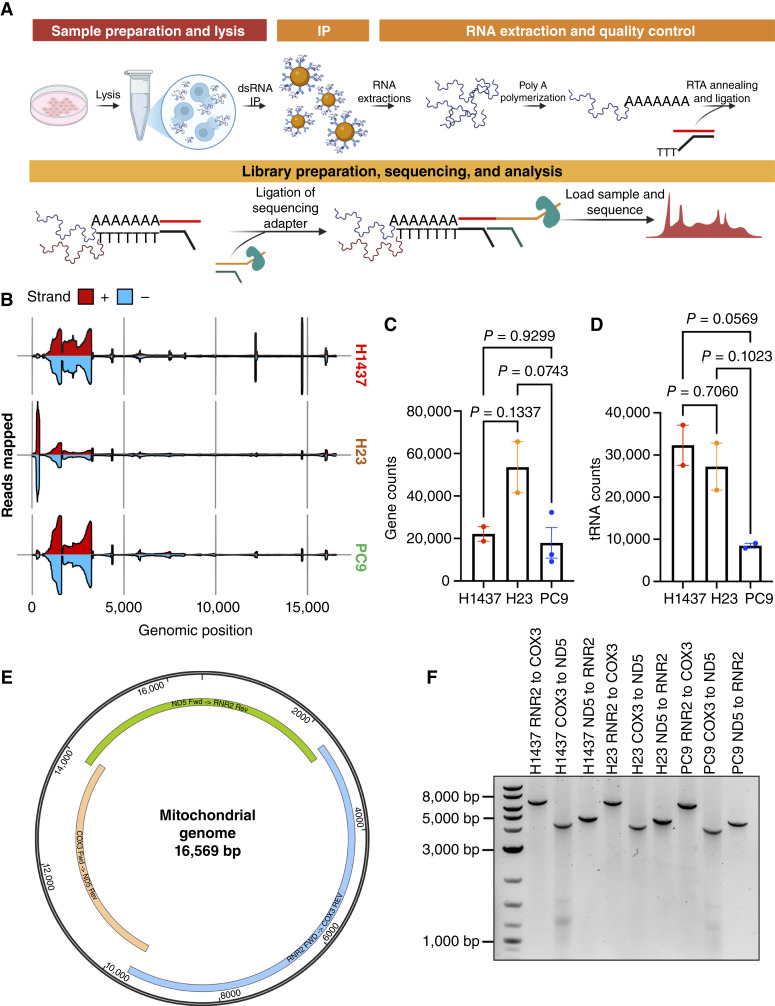
Enrichment of mtdsRNAs is captured through dsRNA-seq in selected NSCLC cell lines. **A,** Workflow of RIP-seq. **B,** Quantification of unique reads detected by RIP-seq. Reads from the heavy (red) and light (blue) strands were mapped to the mitochondrial genome ChrMT. See Supplementary Fig. S1 for an enlarged view of the sequencing data. **C** and **D,** Statistical analysis of successfully aligned reads per genomic feature (gene, CDS, rRNA, exon, and D-loop, with either tRNA included or excluded). Bar represents average. Error bars represent ± SEM. *P* values are represented as is. One-way ANOVA was completed. Each dot represents an independent experiment (*n* = 3). **E,** Mitochondrial genome mapped with primers used for long-read sequencing. Primers can be found in Supplementary Table S1. Green: ND5 Fwd → RNR2 Rev. Blue: RNR2 Fwd → Cox3 Rev. Orange: Cox3 Fwd → ND5 Rev. **F,** Gel showing the bands generated by PCR across three different cell lines used for ADAR sequencing. Representative of three independent experiments (*n* = 3).

To validate the presence and the abundance of mtdsRNAs, we performed dsRNA immunoprecipitation using the J2 monoclonal antibody specific for dsRNA (3), followed by sequencing (RIP-seq) using the Oxford Nanopore long-read sequencing technique as previously described (Fig. 2A; ref. 5). J2 antibody recognizes mtdsRNA that is approximately 40 to 50 bp in length across mammalian species (23). To validate the stratification into high, medium, and low mtdsRNA groups, we selected representative LUAD cell lines from each group and performed sequencing. RIP-seq revealed varying levels dsRNA from both H- (red) and L- (blue) strands across the three cell lines (Fig. 2B; Supplementary Fig. S1). Interestingly, H23, the representative cell line from the intermediate group, displayed the highest mtdsRNA signal. Most abundant mtdsRNAs aligned to the ribosomal (R) RNAs and displacement- (D-) loop regions on average (Supplementary Tables S2 and S3). Quantification of the recovered dsRNA from total RNA extracted further confirmed the differential mtdsRNA levels across the cell lines (Fig. 2C and D). RIP-seq reads were successfully mapped to ChrMT (Supplementary Fig. S1), with H1437 (Average = 17.46%) having the highest proportion of mapped reads compared with H23 (Average = 4.81%) and PC9 (Average = 3.98%; Supplementary Table S4). To validate the mitochondrial origin of dsRNA detected by J2 RIP-seq, we performed parallel RIP-seq analysis in cells treated with the mitochondrial transcription inhibitor, IMT-1, as a negative control (Supplementary Fig. S2; refs. 3, 24). IMT-1 treatment markedly reduced ChrMT mapped J2 RIP-seq reads, supporting the mitochondrial origin of the dsRNA species (Supplementary Fig. S2). This further supports our *in silico* analysis in which PC9 had the lowest predicted mtdsRNA values. Statistical analysis of the aligned reads reveals only a significant enrichment in H23 in comparison with H1437 and PC9 (Fig. 2C and D).

To determine whether the mtdsRNA detected was exported into the cytoplasm, we investigated the presence of ADAR1-driven editing. ADAR1 converts adenosine (A) to inosine (I) in dsRNA and is detected based on mismatches in the genome (25). As an initial assessment of spatial proximity between ADAR1 and dsRNA, we performed immunofluorescence analysis to examine colocalization between ADAR1 and dsRNA detected by J2 (Supplementary Fig. S3). Across cell lines examined, ADAR1 and J2 had minimal spatial overlap between the two signals. This lack of colocalization suggested that ADAR1 interaction with dsRNA may be transient or precede the formation of J2-detectable dsRNA structures. Given the absence of colocalization by imaging, we next used sequencing-based approach to sensitively assess ADAR1 engagement with mtdsRNA. RIP-seq analysis identified ADAR1-dependent RNA-editing events, defined by reproducible A-to-I substitutions enriched within the mtdsRNA regions. We next sought to confirm that these signals were not attributable to underlying DNA sequence alterations by performing long-read PCR at the DNA level (Fig. 2E and F). Long-read sequencing demonstrated concordance with the reference sequence, thereby supporting the conclusion that the observed discrepancies at the RNA level arise from ADAR-mediated editing rather than genomic variations, including mitochondrial heteroplasmy (Supplementary Fig. S4A).

**Figure 3.**
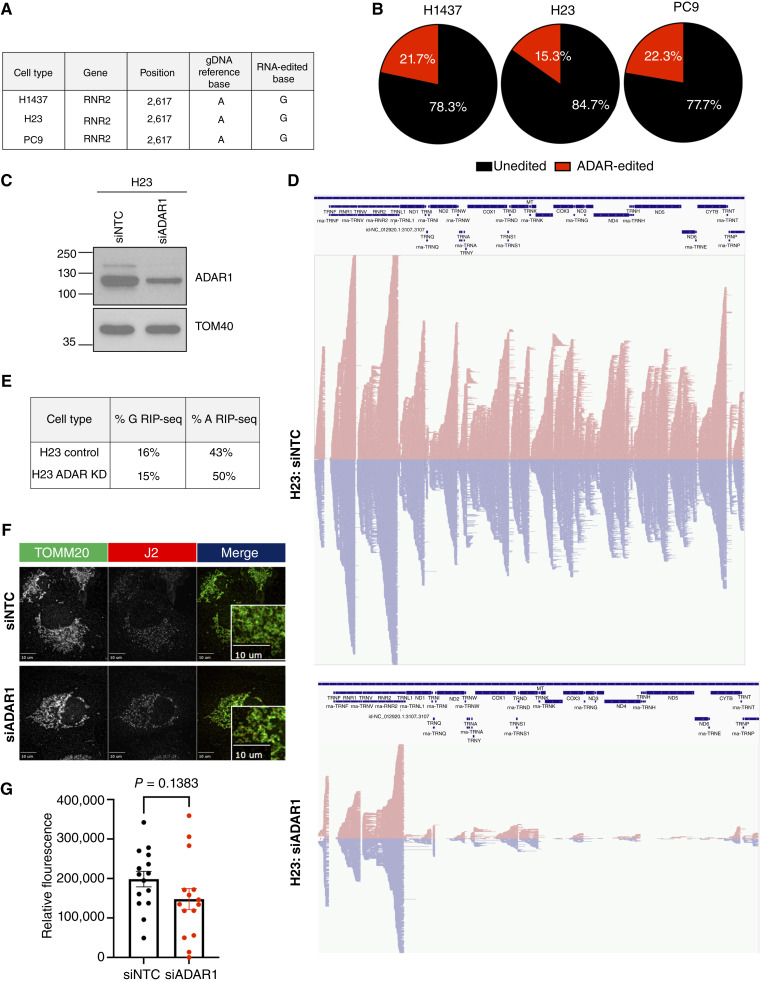
mtdsRNAs exhibit low levels of ADAR1 editing. **A,** Identification of a common ADAR-editing sites in different cell lines. **B,** Pie chart of ADAR-edited (red) and unedited (black) read percentages at position 2,617 in RNR2 detected across H1437, H23, and PC9. **C,** Western blot imaging of ADAR1 and TOMM20 (loading control) in H23. Representative of three independent experiments (*n* = 3). **D,** Representative histogram of reads mapped to the mitochondrial genome (top) in H23 treated with siRNA targeting nothing (siNTC) or ADAR1 (siADAR1). **E,** Quantification of % G and A sites detected in H23 treated with siRNA targeting nothing (siNTC) or ADAR1 (siADAR1) from RIP-seq. **F,** Representative immunofluorescence imaging of H23 following siRNA-mediated depletion of ADAR or nothing (siNTC) at 40× organized from the highest mtdsRNA-predicted value to the lowest. TOMM20 (green), mitochondrial marker, J2 (red), mtdsRNA marker. PCC is displayed as an average across three independent experiments (*n* = 3). **G,** Quantification of immunofluorescence detected. Bar represent average. Error bars represent ± SEM. *P* values are represented as is. Each dot represents a cell from three independent experiments (*n* = 3).

Across these cell lines, we have only identified position 2,617 in RNR2 to be edited prevalently across three independent experiments (Fig. 3A). Of the mtdsRNAs detected, 21.7%, 15.3%, and 22.3% of position 2,617 were edited across H1437, H23, and PC9, respectively (Fig. 3B). Additional sites of ADAR editing were identified but were localized to the tRNAs and were not replicated across different biological replicates. These editing events suggest that they may represent transient or unstable editing events. The limited number of stable, high-frequency editing site is notable and suggests that most ADAR1-mediated editing events on mtdsRNA may be rapidly degraded and therefore not readily detectable at steady state.

To assess whether ADAR1-associated RNA editing reflects engagement with mtdsRNA, we performed siRNA-mediated knockdown of ADAR1 in H23 cells (Fig. 3C), which exhibited the highest mtdsRNA abundance among the cell lines analyzed. RIP-seq analysis revealed a reproducible shift in nucleotide composition of ADAR1-associated RNA following ADAR1 knockdown (Fig. 3D and E). Specifically, compared with nontargeting control (siNTC), depletion of ADAR1 (siADAR1) resulted in a modest reduction of guanosine (G) content accompanied by a marked increase in adenosine (A) content within the captured RNA population (Fig. 3E). This reciprocal change in A and G content is consistent with reduced A-to-I editing activity upon ADAR1 depletion, as inosine is detected as guanosine during sequencing. These findings indicate that ADAR1 knockdown dampens RNA editing at a global level, even in the absence of pronounced changes at individual high-frequency editing sites.

We then tested whether ADAR1-mediated editing influences the abundance of J2 detectable mtdsRNA by assessing dsRNA abundance following ADAR1 knockdown by immunofluorescence. Quantitative analysis of J2 signal revealed no significant differences following siRNA-mediated ADAR depletion (Fig. 3F and G). This indicates that depletion of ADAR1 does not alter steady-state mtdsRNA abundance as detected by J2, further supporting J2 immunoreactivity as a direct and quantitative measure of mtdsRNA burden, independent of ADAR1-mediated editing.

In addition, to exclude the possibility that these RNA-level discrepancies were attributed to underlying DNA sequence alterations, including mitochondrial heteroplasmy (Supplementary Fig. S4B), we looked at alleles at the site of interest. Together, these findings demonstrate that mtdsRNA is not only present but amounts vary significantly across lung cancer cell lines, and ADAR1-associated editing is limited, site-selective, and likely constrained by rapid turnover of edited dsRNA species.

### mtdsRNA abundance is stratified into two cohorts

**Figure 4.**
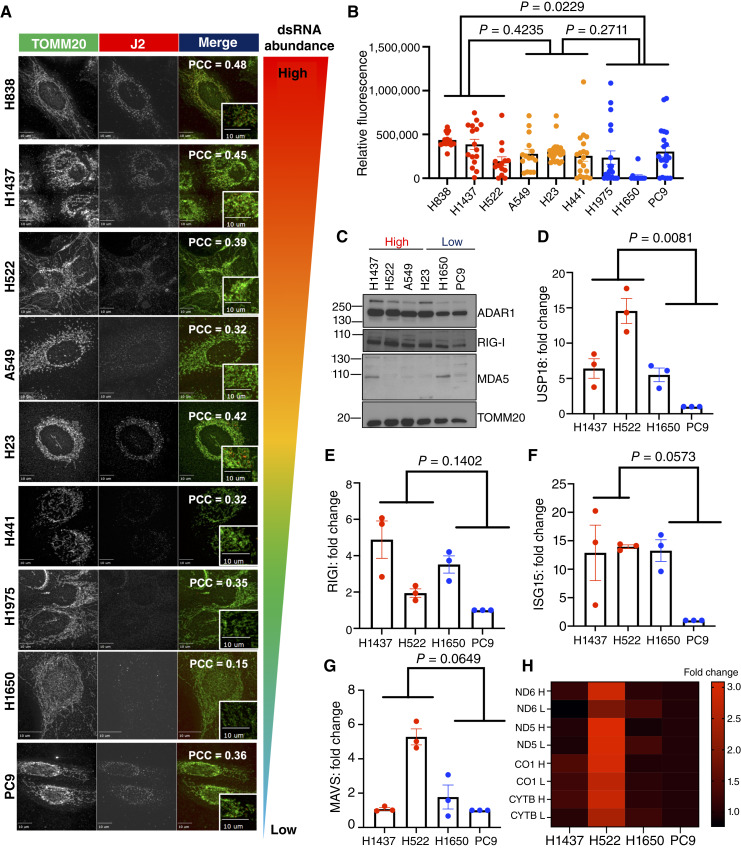
mtdsRNAs do not activate IFN-I signaling pathways. **A,** Representative immunofluorescence imaging at 40× organized from the highest mtdsRNA-predicted value to the lowest. TOMM20 (green), mitochondrial marker, J2 (red), mtdsRNA marker. PCC is displayed as an average across three independent experiments (*n* = 3). **B,** Quantification of immunofluorescence detected. Bar represent average. Error bars represent ± SEM. *P* values are represented as is. Each dot represents a cell from three independent experiments (*n* = 3). **C,** Western blot imaging of ADAR1, RIG-I, MDA5, and TOMM20 (loading control) in cell lines with high (red) and low (blue) levels of mtdsRNA. Representative of three independent experiments (*n* = 3). **D–G,** Relative RT-qPCR values of USP18, ISG15, RIG-I, and MAVS. Normalized to PC9. GADPH was used as a house keeping gene. Fold change shown normalized to PC9. Bar represent average. Error bars represent ± SEM. *P* values are represented as is. Representative of three independent experiments (*n* = 3). **H,** Strand-specific RT-qPCR across mitochondrial genes. Normalized to PC9. GADPH was used as a house keeping gene. *N* = 3.

Having established by RIP-seq analysis that mtdsRNA abundance varies across NSCLC models, we next extended these observations at a larger scale by experimentally confirming mtdsRNA levels derived from *in silico* analysis. To enable an initial, unbiased stratification across multiple models, we first leveraged previously quantified mitochondrial H- and L-strand transcript levels derived from transcriptomic analyses (Fig. 1). This *in silico* approach served as a hypothesis-generating framework to predict relative mtdsRNA abundance. Based on these predictions, three representative NSCLC cell lines were chosen from each group (high, medium, and Low) and assessed mtdsRNA levels by immunofluorescence using dsRNA-specific staining with the J2 antibody (Fig. 4A and B; Supplementary Fig. S5). Across the panel of cell lines, we observed varying levels of enrichment of J2-positive puncta, indicating differences in mtdsRNA abundance. Quantification of the relative J2 fluorescence intensity revealed a clear stratification of mtdsRNA levels amongst the cell lines. A pronounced reduction in J2 puncta was observed in cell lines predicted to have low mtdsRNA abundance compared with those in the high-mtdsRNA group, supporting concordance between transcriptomic inference and direct J2-based predictions. In contrast, the differences in J2 signal intensity amongst the medium group were not statistically significant when compared with either the high or the low group. These findings suggest that mtdsRNA abundance can be broadly stratified into two distinct categories: high and low cytoplasmic mtdsRNA.

To assess the subcellular distribution of mtdsRNA, we quantified the spatial relationship between J2 signal and mitochondrial markers using PCC. Across the profiled cell lines, the average PCC value (26) was 0.37, indicating limited colocalization between J2 and mitochondrial markers. To further evaluate compartmental separation between mitochondria and cytosolic dsRNA-interacting machinery, we also assessed ADAR1, a well-characterized cytosolic dsRNA editor with mitochondrial markers. ADAR1 exhibited minimal co-localization with mitochondria as reflected by a low average PCC value 0.07 across the cell lines (Supplementary Fig. S6). Together, these analyses indicate that a substantial fraction of the J2 signal is not associated with the mitochondria and is consistent with the presence of a dsRNA pool that is accessible to nonmitochondrial, including cytosolic, RNA interacting machinery.

### Presence of endogenous mtdsRNA does not activate IFN-I pathways

The presence of mtdsRNA has been shown to canonically drive the IFN-I response (3, 5, 27). IFN-I signaling is a rapid and transient immune pathway that is typically triggered by a viral infection or presence of DAMPs (3). In this study, we explored whether endogenously enriched mtdsRNA could drive constitute rewiring of IFN-I–associated proteins, leading to sustained upregulation of IFN-I signaling pathways. First, using the same *in silico* stratification based on mitochondrial H- and L-strand transcript levels, we analyzed the expression of a curated panel of IFN-stimulated genes (ISG) and IFN pathway components (Supplementary Fig. S7). Here, we observed no consistent upregulation or repression of IFN-related genes associated with mtdsRNA levels. Whereas individual genes exhibited variability across models, no coherent IFN-I transcriptional signatures emerged that correlated with predicted mtdsRNA abundance. These findings suggest that variation in mtdsRNA abundance across NSCLC cell lines is not sufficient, in isolation, to drive a basal IFN response at the transcript level.

To confirm our *in silico* analysis at a protein level, we assessed the expression of key IFN-I–related proteins in six cell lines from the high- and low-mtdsRNA cohorts. As expected, we did not observe consistent changes in protein expression across these cell lines (Fig. 4C). We further evaluated IFN-I transcript expression using RT-qPCR (Fig. 4D–G). H1437 and H522 were selected as representatives of the high-mtdsRNA group, and H1650 and PC9s of the low-mtdsRNA group, based on the largest differences observed in both J2 immunofluorescence signal and ADAR1/RIG-I protein levels. Whereas loss of PNPT1, a critical regulator of mtdsRNA, has been shown to activate signaling (3, 5, 27), we questioned whether endogenous mtdsRNA alone could regulate IFN-I–related genes in the absence of genetic perturbation. Transcript levels of canonical ISGs, including ISG15, USP18, RIG-I, MDA5, and MAVS, were monitored across multiple LUAD cell lines (Fig. 3D–G). Although expression levels varied across the panel, only USP18 was significantly enriched in high mtdsRNAs cell lines. Strand-specific RT-qPCR (28) also revealed no significant differences (Fig. 4H), though a trend toward enrichment in IFN-1–related genes were observed in high-mtdsRNA lines (Fig. 3G). Collectively, these results indicate that whereas mtdsRNA is detectable in NSCLC cells, its endogenous accumulation is generally insufficient to elicit a robust IFN-I response.

We then assessed pathway engagement at the subcellular level by immunofluorescence analysis of MAVS localization relative to the mitochondria (Supplementary Fig. S8). Across the cell lines profiled, MAVS exhibited comparable mitochondrial localization with no evidence of enhanced clustering or redistribution of MAVS indicative of pathway activation. Together, these data demonstrate that differences in mtdsRNA abundance across NSCLC cell lines are not associated with constitutive activation of MAVS or basal IFN-I signaling, suggesting that additional regulatory mechanisms constrain innate immune activation despite the presence of mtdsRNA. These findings suggest that NSCLC cells may tolerate elevated mitochondrial mtdsRNAs by limiting its cytosolic sensing, potentially adapting their antiviral signaling pathways to maintain cellular fitness.

### mtdsRNA is identified across a broad spectrum of human cancers

**Figure 5.**
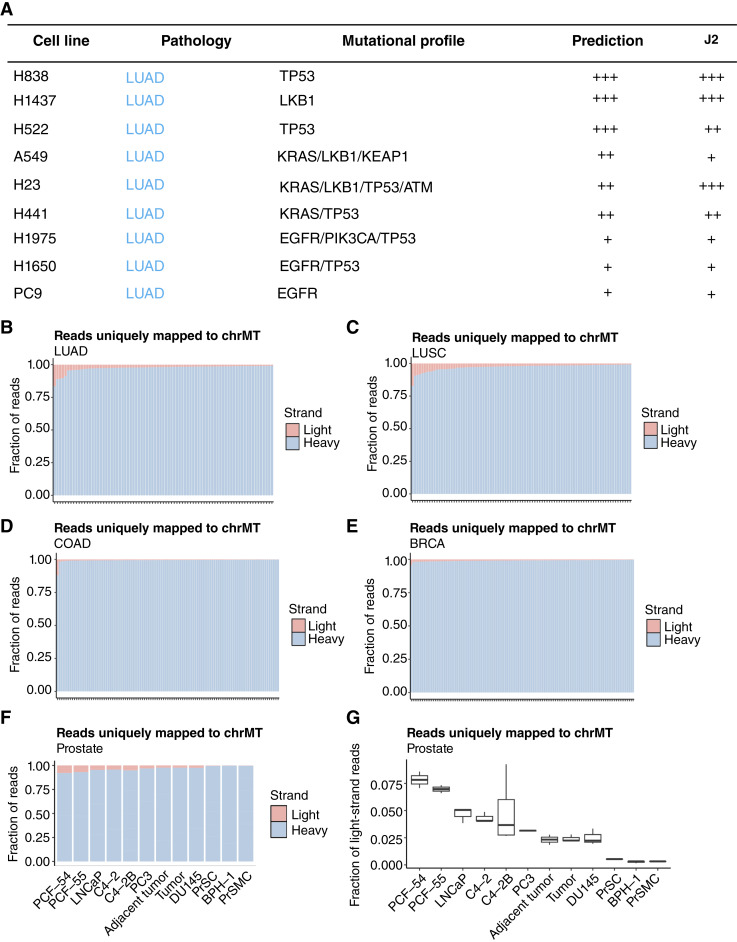
mtdsRNA is identified beyond NSCLC cell lines. **A,** Summary table of the results. **B** and **C,** Fraction of reads mapped to light (red) and heavy (blue) strands of mitochondrial genome (ChrMT) detected in LUAD and LUSC patient samples. Each bar represents one patient. **D** and **E,** Fraction of reads uniquely mapped to light (red) and heavy (blue) strands of mitochondrial genome (ChrMT) in COAD and breast cancer (BRCA) patient samples. Each bar represents one patient. **F,** Fraction of reads mapped to light (red) and heavy (blue) strands of mitochondrial genome (ChrMT) detected in prostate cancer cell lines and patient samples. **G,** Box plot of fraction of reads mapped to the light strand of chrMT in prostate cancer cell lines and patient samples.

Our *in vitro* analysis revealed that mtdsRNA abundance varies significantly across LUAD cell lines and correlated with differential expression of select ISGs. However, basal levels of mtdsRNA alone were insufficient to consistently activate IFN-l response. Importantly, despite the lack of IFN-I activation, J2-based detection provided a robust and quantitative measure of mtdsRNA abundance across cell lines, enabling reliable stratification independent of downstream immune signaling (Fig. 5A). These findings motivated us to investigate whether mitochondrial transcript abundance patterns, and consequently mtdsRNA generation, vary across NSCLC patient samples and in different tumor types. Thus, we analyzed stranded RNA-seq datasets from patients with cancer generated by the CPTAC (29). A small fraction (∼5%) of reads mapped to the light strand of the mitochondrial genome in LUAD (Fig. 5B) and LUSC (Fig. 5C). Interestingly, some patients exhibit a higher fraction (10%∼15%) of reads mapping to the light strand (Fig. 5B and C). We further explored the available data from patients with COAD and breast cancer from the CPTAC. One patient with COAD showed 12% of reads mapping to the light strand (Fig. 5D). Less than 2% of reads mapped to the light strand in patients with breast cancer (Fig. 5E). We also analyzed publicly available stranded RNA-seq datasets from prostate cancer cell lines (30–32). On average, 5% of mitochondrial reads mapped to the light strand (Fig. 5F and G). Cell lines PCF-54 and PCF-54 exhibited a higher proportion, approximately 7.5% (Fig. 5G). These findings suggest that abundance of transcripts of the mitochondrial light strand, a potential source of mtdsRNA, varies across cancer types and patient samples. With elevated levels of subsets of mtdsRNA in prostate and lung cancers, our findings highlight a tumor-specific difference in mitochondrial transcript abundance that may influence mtdsRNA-mediated immune signaling in cancer types beyond NSCLC.

## Discussion

In this study, we provide a comprehensive analysis of mtdsRNA across a diverse panel of NSCLC cell lines. Using publicly available stranded RNA-seq datasets, we identified a subset of cell lines with unexpectedly high levels of mitochondrial L-strand transcripts, suggesting the potential formation of mtdsRNA and failed degradation. This phenomenon was substantiated through RIP-seq, followed by long-read sequencing, strand-specific mapping, and quantification of cytoplasmic dsRNA. Collectively, these results demonstrate that mtdsRNA abundance varies widely across NSCLC models and can reach moderate levels that may induce an IFN-l response.

The mtdsRNA transcripts are difficult to identify in the public RNA-seq databases due to a combination of biological and technical challenges (33, 34). One reason is that standard RNA-seq library preparation methods and approaches are optimized for cytosolic RNA rather than mitochondrial RNA, resulting in inefficient recovery and underrepresentation of mtdsRNA in sequencing datasets (35). In support of this, mitochondrial RNAs typically lack polyA tails or have shortened polyA tails (36). The mtdsRNAs for the rRNAs were also most abundant, and rRNAs are often selectively depleted in RNA-seq library preparation. Similarly, bioinformatics and computational tools and reference annotations are not always optimized for mitochondrial transcripts, and reliable reference genes for expression normalization are lacking (37). Finally, transcripts from the mitochondrial genome may be ignored, in favor of transcript analysis from the nuclear genome. Diederichs and colleagues revolutionized RNA capture (19) as their RNA preparation differed in that they depleted rRNAs and then sequenced the remaining RNA of 60 different NSCLC cell lines, consequently capturing circular RNAs. This technique, to our interest, yielded abundant L-strand transcripts in NSCLC cell lines, which served as an indicator of mtdsRNAs. Here, we used the H23 cell line as the “bait” to search for L-strand transcripts, which we had previously shown had abundant mtdsRNAs (5). We subsequently used their public RNA-seq datasets to profile the reads for L-strand transcripts across NSCLC cell lines.

Our long-read sequencing results were resolved at nucleotide resolution. This approach confirmed concordance with the mitochondrial reference genome and attributed RNA-level discrepancies to ADAR-mediated editing. Notably, position 2,617 in RNR2 was consistently edited across multiple NSCLC cell lines. The reproducibility and quantifiable nature of this site-specific editing suggest that it could serve as a potential molecular signature of mtdsRNAs and potentially reflect mitochondrial stress states or dysregulated RNA processing. These findings raise a possibility that site-specific mtdsRNA editing could be developed as a diagnostic or stratification biomarker, particularly if detected in patient-derived tumor biopsies or circulating biofluids. However, position 2,617 is modified by the mitochondrial tRNA methyltransferase TRMT61B to give an m1A modification (38, 39), which stabilizes interactions within the mitoribosomes (40). Thus, confirmation of ADAR editing at this site requires careful analysis, particularly as future studies may consider whether nucleotide 2,617 of RNR2 correlates with tumor subtypes and tumor metabolic status.

Despite detectable accumulation of mtdsRNA, our data reveal that it does not consistently lead to activation of IFN-I signaling, a striking departure from previous studies that reported robust IFN-I activation following mtdsRNA buildup (3, 5). This suggests that endogenous accumulation of mtdsRNA alone, in the absence of mitochondrial stress or RNA export defects, may be insufficient to surpass the activation threshold for innate immune sensing and may contribute to alternative stress mechanisms that have yet to be explored. The apparent tolerance of NSCLC cell lines to high mtdsRNA levels raises key questions about how cancer cells avoid deleterious immune activation despite harboring immunostimulatory dsRNA. Sequestration of mtdsRNA into compartments or bound by RNA-binding proteins that mask it from recognition by cytosolic pattern recognition receptors (PRR) such as MDA5 and RIG-I have been previously noted can provide some speculations to our observations (41). In addition, proteins like LRPPRC/SLIRP known to regulate mitochondrial RNA stability and processing (42–44) may limit the cytosolic availability of unprocessed or hybrid RNA species. Enhanced ADAR-editing pathways may also efficiently tamp a potential IFN-l signal (45).

Mitochondrial quality control pathways, including mitophagy and autophagy, may additionally contribute to minimal activation of IFN-I signaling. Mitophagy and autophagy canonically contribute to the clearance of damaged mitochondria and potentially dsRNA-containing mitochondrial fragments before they engage innate immune sensors (46, 47). Mitophagy selectively removes dysfunctional mitochondria and helps restrain mitochondrial stress derived signals that can otherwise activate inflammatory signaling (48, 49). In addition, autophagy has been implicated in the degradation of components of RIG-I-like receptor signaling, providing a negative regulatory influence on innate immune activation (49). These mechanisms collectively may diminish the availability of cytosolic dsRNA for PRR recognition and help explain why cytosolic dsRNA alone does not universally trigger IFN-I response in cancer cells.

Alternatively, tumor cells have also reprogrammed their innate immune threshold to selectively avoid chronic IFN-I signaling due to its antiproliferative and immunostimulatory effects. Previous reports have shown that chronic IFN-I exposure leads to epigenetic silencing of key pathway components such as IRF7 and STING or posttranslational inactivation of dsRNA sensors (50, 51). In addition, mutations in *PNPT1* result in mitochondrial and autoimmune diseases (11, 52–55), but patients tolerate elevated levels of mtdsRNA, often surviving until late in life (9, 10). Together, these observations support a model in which cytosolic dsRNA accumulation alone is not sufficient to elicit immune activation, notably in cancer cells, that have consistently evolved multiple layers of stress tolerance, including enhanced RNA degradation, sequestration, and organelle quality control pathways. Therefore, elevated mtdsRNA might persist intracellularly without triggering a full immune response due to tumor acquired defects or adaptations in sensing machinery.

Our extended analysis to clinical tumor datasets has revealed that L-strand transcripts, a proxy for potential mtdsRNA production varies not only within NSCLC but across tumor types. LUAD and prostate cancer samples displayed elevated light-strand transcript in a subset of tumors, whereas breast cancer showed consistently low levels. This divergence may reflect differences in mitochondrial transcripts across different tumor tissue types as exemplified by differentially expressed genes such as mitochondrial transcription factor A (TFAM), TFB2M, and POLRMT, a key regulator of mitochondrial RNA synthesis and immune evasion in cancer (56–58). Furthermore, copy-number variation of mtDNA or mutations in the D-loop promoter region may shift transcriptional dynamics toward asymmetric transcription of light versus heavy strands. These tumor-specific mitochondrial programs may influence the capacity for dsRNA formation and thus alter the innate immune profile of a tumor. It is also notable that LUAD and prostate cancer are both tumors in which mitochondrial metabolism plays a prominent role in progression and therapy resistance. Prostate cancer relies heavily on oxidative phosphorylation and mitochondrial biogenesis (59, 60). These metabolic dependencies may inadvertently elevate transcript output and increase the likelihood of dsRNA formation, especially under metabolic stress or hypoxic conditions. However, given our observation, we posit that the elevated transcript output alone will not trigger an immunomodulatory role and will require a direct manipulation of the mtdsRNA export machinery for patient benefit.

Finally, our findings highlight the utility of J2 antibody as a readout of intracellular dsRNA burden, independent of genomic profiles. Thus, the detection of dsRNA through an antibody opens new possibilities for stratifying tumors based on RNA metabolism. However, the translation of J2-based profiling into clinical workflows presents several challenges (61). Although J2 is widely used to detect dsRNA, its specificity and sensitivity can vary with dsRNA length and composition, and alternative clones may outperform J2 in formalin-fixed tissues (62). Standardization of sample handling, quantification thresholds, and signal interpretation will be critical before J2-based dsRNA profiling can be integrated into tumor classification or liquid biopsy assays. J2-based profiling may provide a functional readout of intracellular dsRNA burden and can offer a complementary approach for identifying tumors with features associated with latent immune activation, which could be explored in future studies evaluating immune-stimulatory or combinatorial therapeutic strategies.

Taken together, we reveal that mtdsRNA is a pervasive yet variably tolerated feature of NSCLC and other tumors. Whereas it accumulates in the cytoplasm of select cell lines and tumors, it does not inherently drive IFN-I signaling, highlighting the importance of cellular buffering mechanisms such as autophagy, mitophagy, and immune desensitization pathways in shaping the outcome of endogenous dsRNA sensing. These findings underscore a broader paradigm in which mitochondrial transcript heterogeneity contributes to the immunologic identity of tumors but is modulated by tumor-intrinsic tolerance mechanisms. Future studies dissecting the molecular contexts that convert mtdsRNA from inert transcript to immunostimulant ligand may uncover actionable targets for cancer immunotherapy and RNA-based therapeutics.

## Supplementary Material

Supplementary Figure 1 RIP-seq Histogram of H1944, H23 and PC9

Supplementary Figure 2 RIP-seq Histogram of H1944, H23 and PC9 with and without IMT-1 Treatment

Supplementary Figure 3 IF of ADAR1 localization

Supplementary Figure 4 Representative peak analysis for heteroplasmy

Supplementary Figure 5 IF of cell lines with IMT-1 Treatment

Supplementary Figure 6 ADAR1 and MT IF

Supplementary Figure 7 in silico IFN analysis

Supplementary Figure 8 MAVS and MT IF analysis

Supplementary Table 1 Reagents

Supplementary Table 2 RNA read counts analysis

Supplementary Table 3 RNA read counts with tRNA

Supplementary Table 4 Read counts mapped to mitochondrial genome

Supplementary File 1 High, Medium and Low mtdsRNA Quartiles

## Data Availability

The sequencing data generated in this study are publicly available in Gene Expression Omnibus at GSE319333. The remaining data generated in this study are available within the article and its supplementary data files.
