# Supplementary material for: Characterization of Mitochondrial Double-Stranded RNA Levels in Non–Small Cell Lung Carcinoma
Source: Cancer Res Commun. 2026 Apr 7;6(4):769–82. doi: 10.1158/2767-9764.CRC-25-0656 (PMC13054796; doi:10.1158/2767-9764.CRC-25-0656)
Supplement: Supplementary Figure 1 — RIP-seq Histogram of H1944, H23 and PC9 [file crc-25-0656_supplementary_figure_1_suppsf1.pdf]

Supplementary Figure 1: mtdsRNAs sequencing with detailed reads

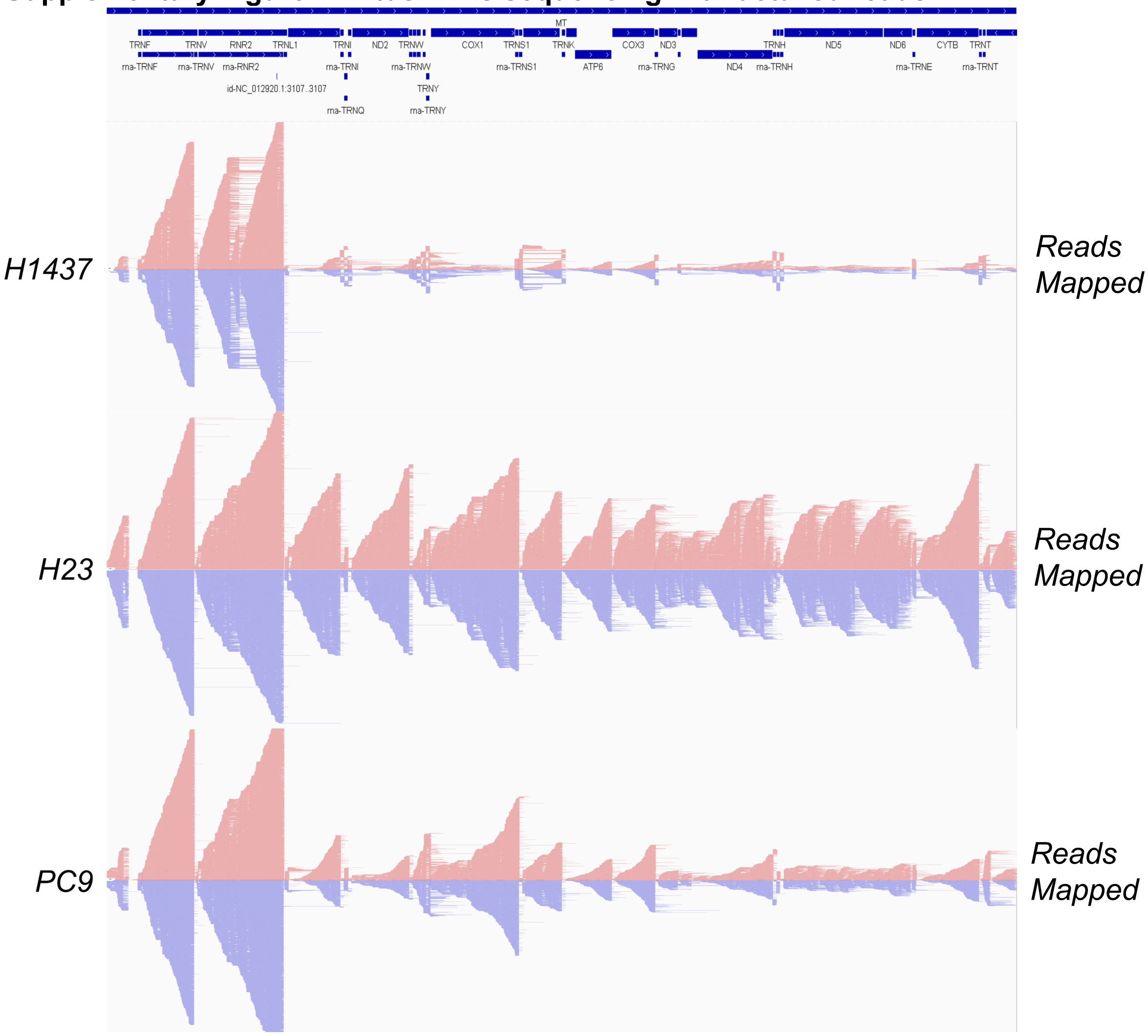

Representative histogram of reads mapped to the mitochondrial genome (top) across three different cell lines (H1437, H23 and PC9).
