# Supplementary material for: Characterization of Mitochondrial Double-Stranded RNA Levels in Non–Small Cell Lung Carcinoma
Source: Cancer Res Commun. 2026 Apr 7;6(4):769–82. doi: 10.1158/2767-9764.CRC-25-0656 (PMC13054796; doi:10.1158/2767-9764.CRC-25-0656)
Supplement: Supplementary Figure 3 — IF of ADAR1 localization [file crc-25-0656_supplementary_figure_3_suppsf3.pdf]

Supplementary Figure 3: ADAR1 does not co-localize with mtdsRNA

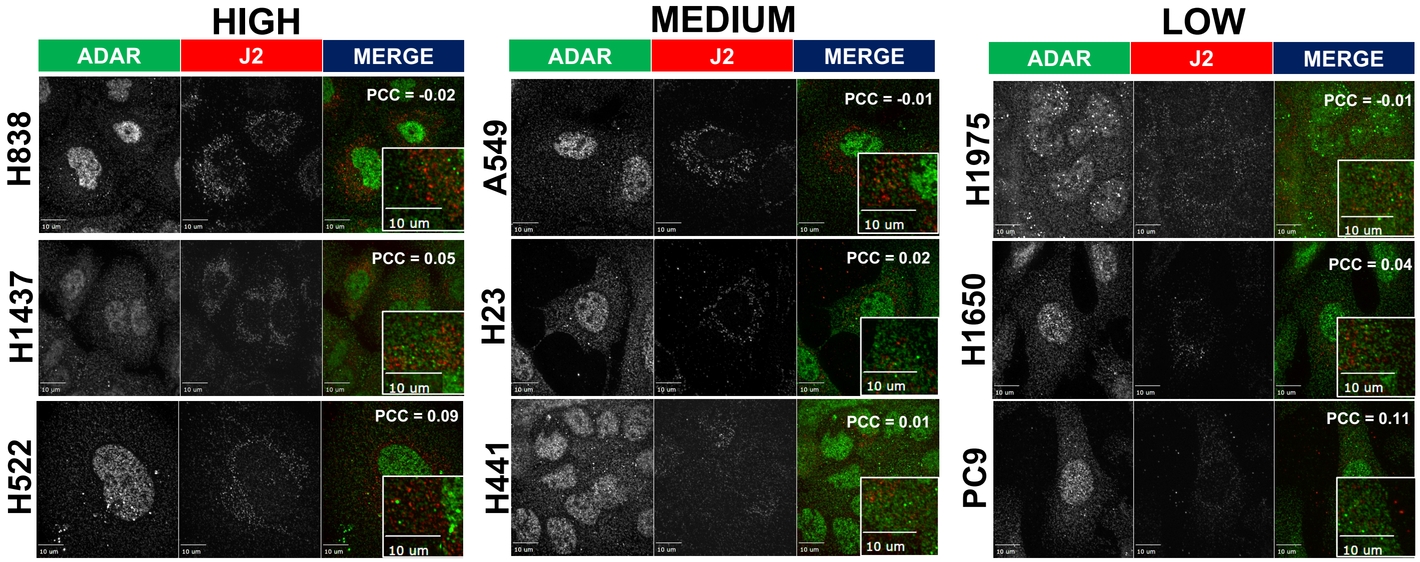

Representative immunofluorescence imaging at 40x organized from highest mtdsRNA predicted value to lowest. ADAR1 (green), mtdsRNA editor marker, and J2 (red), dsRNA marker. Pearson's correlation coefficient (PCC) is displayed as an average across three independent experiments (n=3).
