# Supplementary material for: Characterization of Mitochondrial Double-Stranded RNA Levels in Non–Small Cell Lung Carcinoma
Source: Cancer Res Commun. 2026 Apr 7;6(4):769–82. doi: 10.1158/2767-9764.CRC-25-0656 (PMC13054796; doi:10.1158/2767-9764.CRC-25-0656)
Supplement: Supplementary Figure 4 — Representative peak analysis for heteroplasmy [file crc-25-0656_supplementary_figure_4_suppsf4.pdf]

Supplementary Figure 4: Visualization of the nucleotides at position 2617 of RNR2

A.

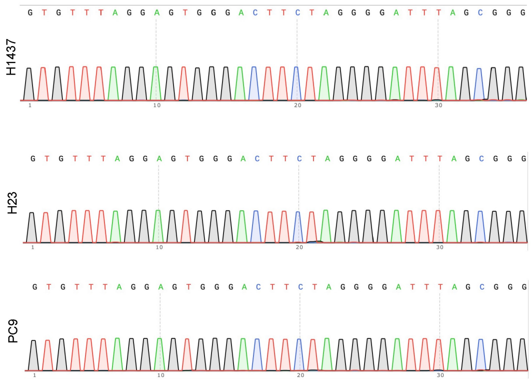

B.

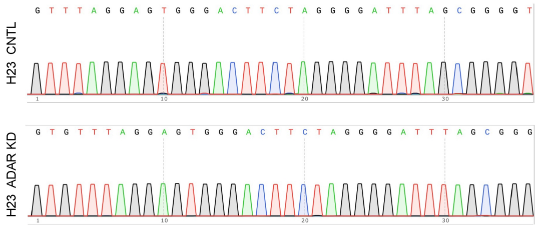

Representative snapshot of integrative genomics viewer (IGV) at position 2617 of RNR2 across three independent sequencing experiments (n=3) in H1437, H23 and PC9 (A) and in H23 treated with siRNA targeting nothing (CNTL) and ADAR1 (ADAR KD).
