# Supplementary material for: Characterization of Mitochondrial Double-Stranded RNA Levels in Non–Small Cell Lung Carcinoma
Source: Cancer Res Commun. 2026 Apr 7;6(4):769–82. doi: 10.1158/2767-9764.CRC-25-0656 (PMC13054796; doi:10.1158/2767-9764.CRC-25-0656)
Supplement: Supplementary Figure 5 — IF of cell lines with IMT-1 Treatment [file crc-25-0656_supplementary_figure_5_suppsf5.pdf]

Supplementary Figure 5: mtdsRNA immunofluorescence following IMT-1 treatment

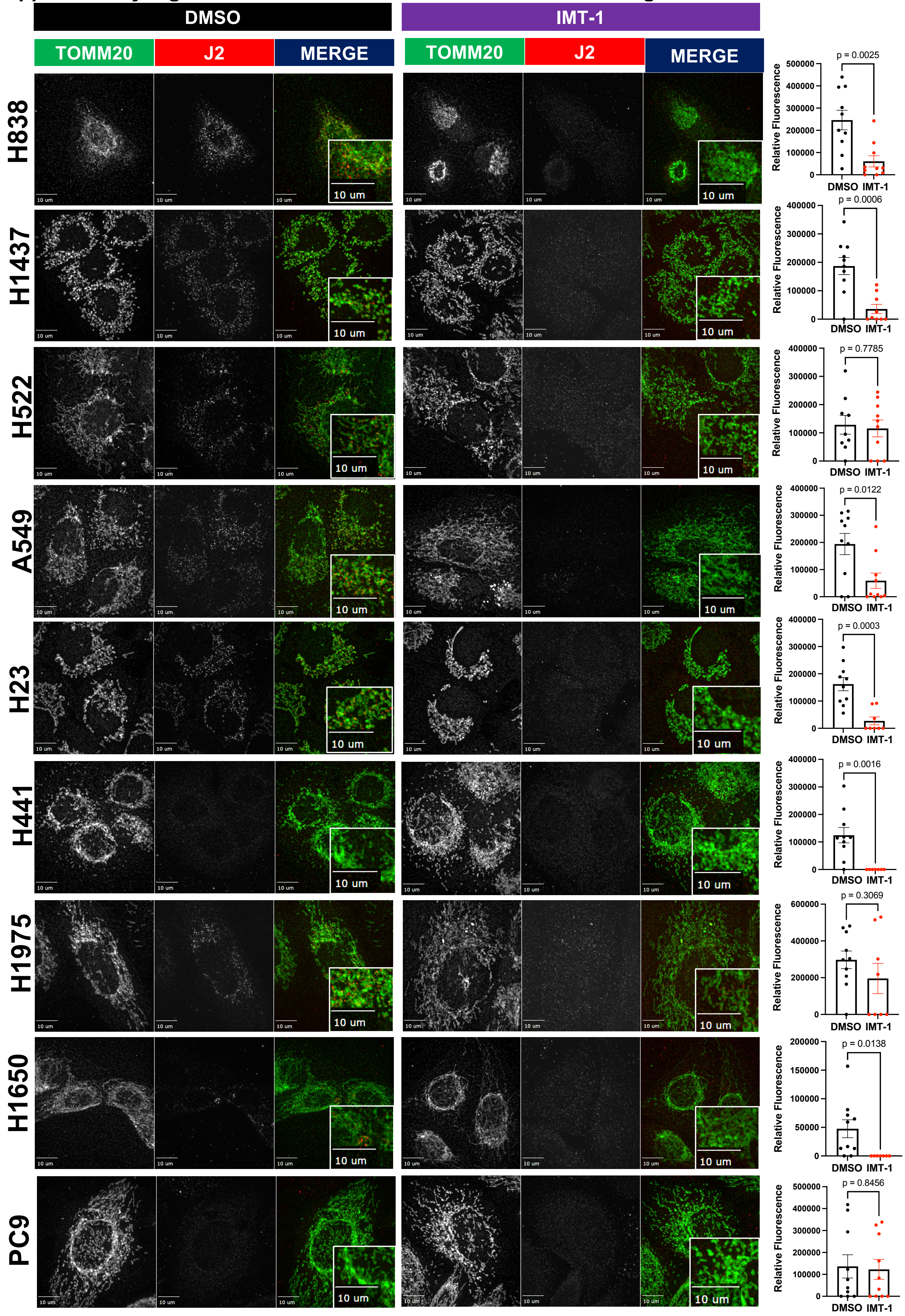

(Left) Representative immunofluorescence imaging at 40x organized from highest mtdsRNA predicted value to lowest. TOMM20 (green), mitochondrial marker, J2 (red), mtdsRNA marker. Pearson's correlation coefficient (PCC) is displayed as an average across three independent experiments (n=3) (Right) Quantification of immunofluorescence detected. Bar represent average. Error bars represent +/- SEM. P values are represented as is. Each dot represents a cell from 3 independent experiments (n=3).
