# Supplementary material for: Characterization of Mitochondrial Double-Stranded RNA Levels in Non–Small Cell Lung Carcinoma
Source: Cancer Res Commun. 2026 Apr 7;6(4):769–82. doi: 10.1158/2767-9764.CRC-25-0656 (PMC13054796; doi:10.1158/2767-9764.CRC-25-0656)
Supplement: Supplementary Figure 6 — ADAR1 and MT IF [file crc-25-0656_supplementary_figure_6_suppsf6.pdf]

Supplementary Figure 6: ADAR1 shows minimal co-localization to the mitochondria

Supplemental Figure 6.

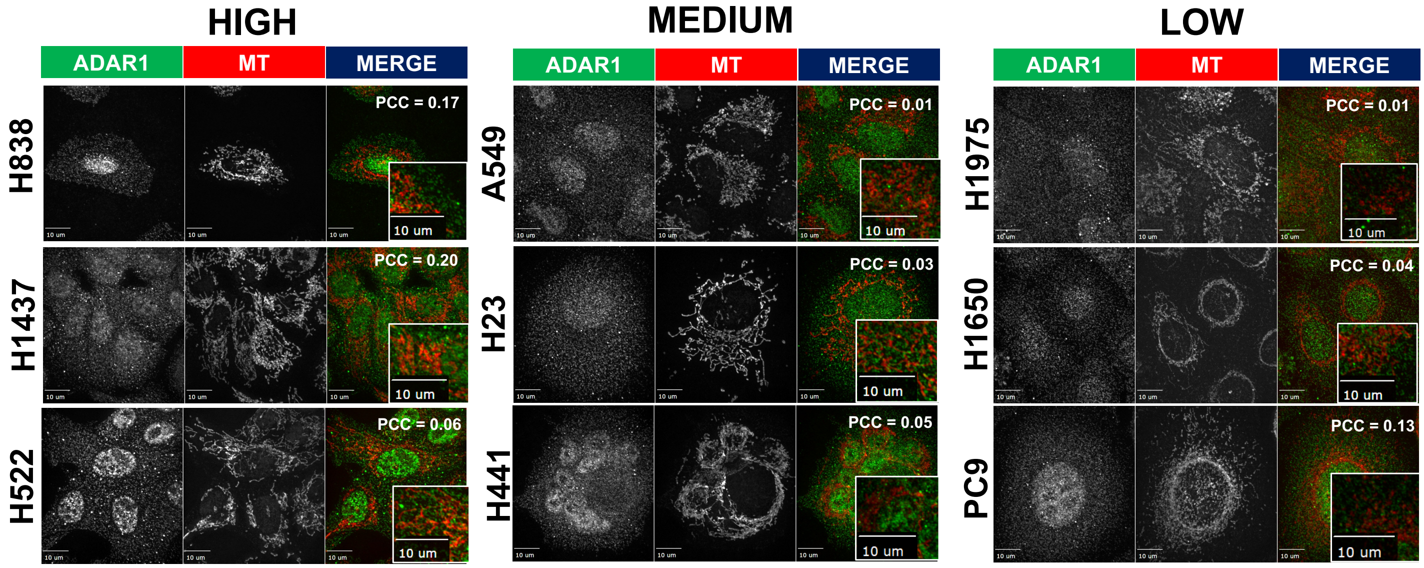

Representative immunofluorescence imaging at 40x organized from highest mtdsRNA predicted value to lowest. ADAR1 (green), mtdsRNA editor marker, and Mitotracker (red), mitochondria marker. Pearson's correlation coefficient (PCC) is displayed as an average across three independent experiments (n=3)
