# Supplementary material for: Characterization of Mitochondrial Double-Stranded RNA Levels in Non–Small Cell Lung Carcinoma
Source: Cancer Res Commun. 2026 Apr 7;6(4):769–82. doi: 10.1158/2767-9764.CRC-25-0656 (PMC13054796; doi:10.1158/2767-9764.CRC-25-0656)
Supplement: Supplementary Figure 7 — in silico IFN analysis [file crc-25-0656_supplementary_figure_7_suppsf7.pdf]

Supplementary Figure 7: IFN-a hallmark gene expression by cell line analysis

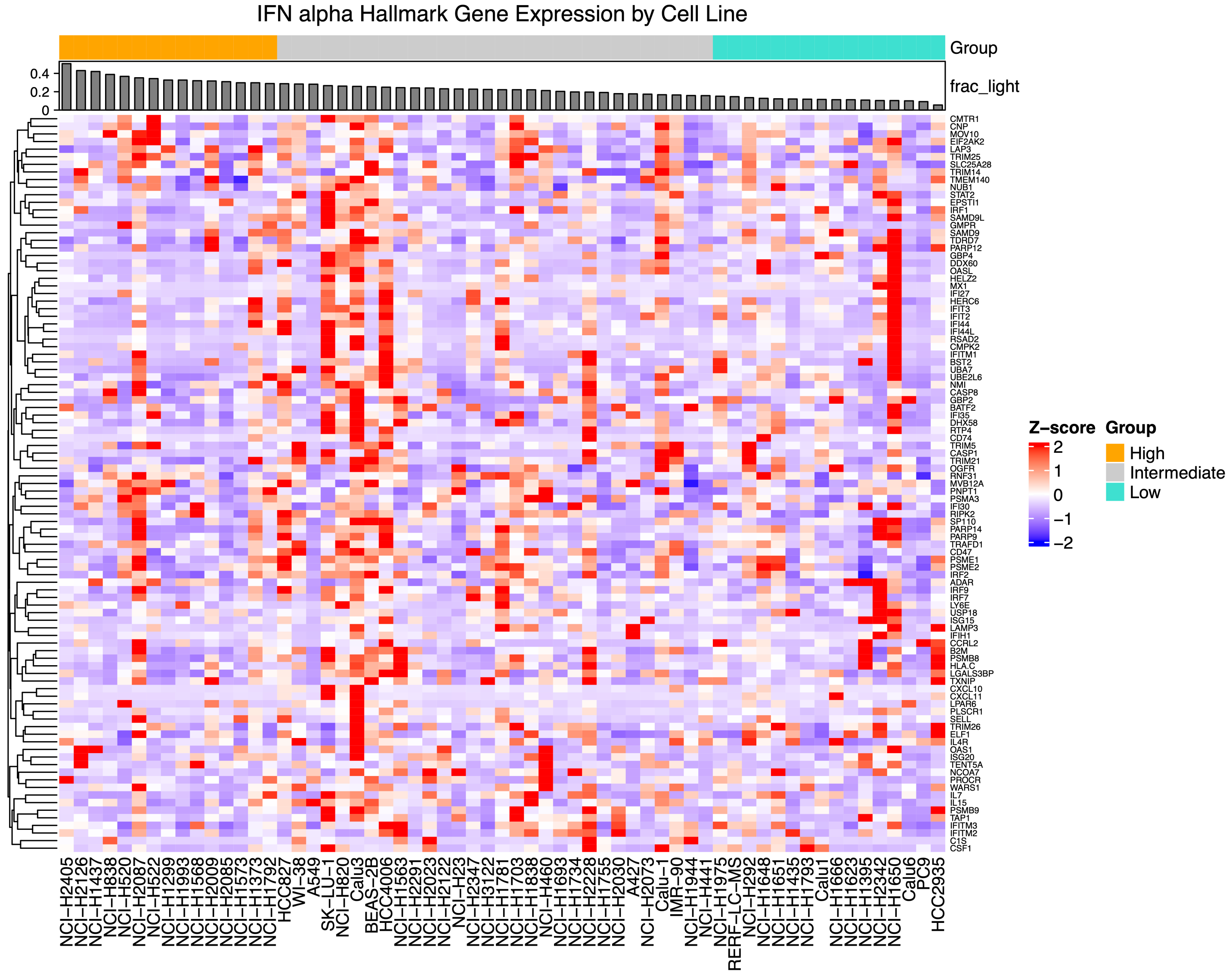

Heatmap of gene expression levels of IFN-1 hallmark genes across 60 lung cancer cell lines. Z-scores of TPM values across cell lines are plotted. Cell lines are grouped into three categories based on their level of fraction of light reads detected at high, medium and low levels.
