# Supplementary material for: Characterization of Mitochondrial Double-Stranded RNA Levels in Non–Small Cell Lung Carcinoma
Source: Cancer Res Commun. 2026 Apr 7;6(4):769–82. doi: 10.1158/2767-9764.CRC-25-0656 (PMC13054796; doi:10.1158/2767-9764.CRC-25-0656)
Supplement: Supplementary Figure 8 — MAVS and MT IF analysis [file crc-25-0656_supplementary_figure_8_suppsf8.pdf]

Supplementary Figure 8: MAVS shows minimal co-localization to the mitochondria

Supplemental Figure 8.

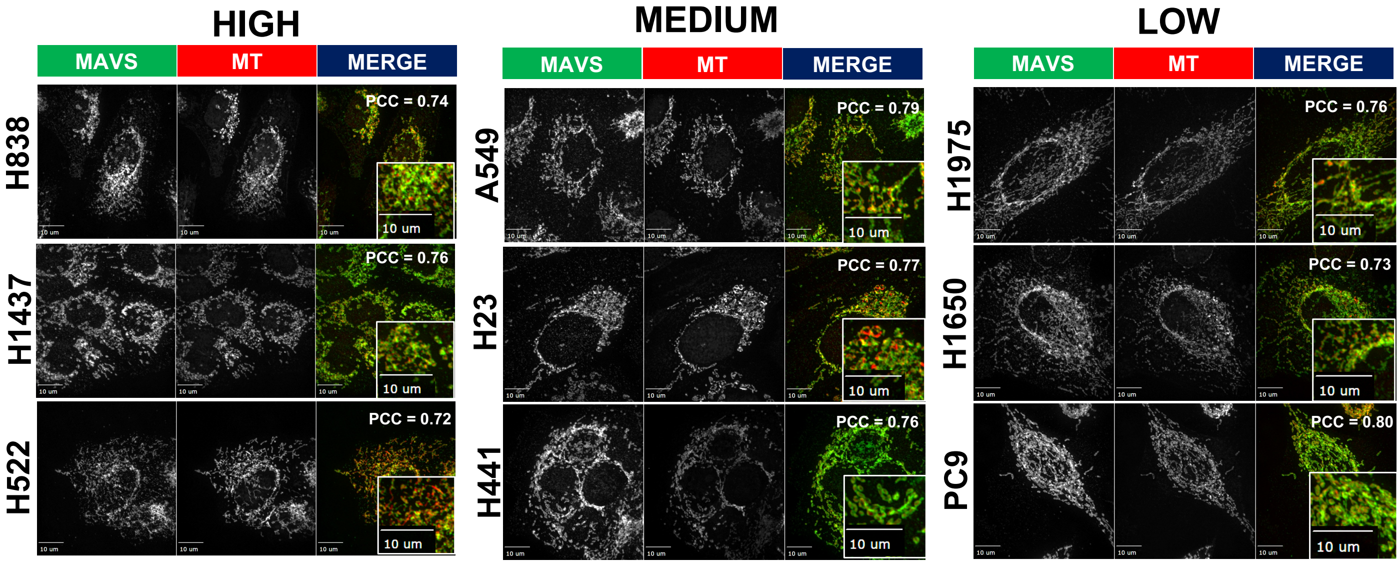

Representative immunofluorescence imaging at 40x organized from highest mtdsRNA predicted value to lowest. MAVS (green), IFN-1 activation marker, and Mitotracker (red), mitochondria marker. Pearson's correlation coefficient (PCC) is displayed as an average across three independent experiments (n=3).
