# Supplementary material for: Characterization of Mitochondrial Double-Stranded RNA Levels in Non–Small Cell Lung Carcinoma
Source: Cancer Res Commun. 2026 Apr 7;6(4):769–82. doi: 10.1158/2767-9764.CRC-25-0656 (PMC13054796; doi:10.1158/2767-9764.CRC-25-0656)
Supplement: Supplementary Table 1 — Reagents [file crc-25-0656_supplementary_table_1_suppst1.docx]

**Supplementary Table 1. Reagents used**

List of all the reagents used in the study

| **Antibodies** | **Vendor** | **Catalog Number** | **Reactivity** | **Concentration** |
| --- | --- | --- | --- | --- |
| MDA5 | Protein Tech | 66770-1-Ig | Mouse | 1:1000 |
| RIG-I | Protein Tech | 67556-1-Ig | Mouse | 1:1000 |
| ADAR1 | Cell Signaling | 14175S | Rabbit | 1:1000 |
| ADAR1 | Abcam | AB307585 | Rabbit | 1:1000 |
| TOMM40 | Protein Tech | 18409-1-AP | Rabbit | 1:1000 |
| J2 | Fisher Scientific | 10010500 | Mouse | 1:500 |
| TOMM20 | Protein Tech | 11802-AP | Rabbit | 1:1000 |
| MAVS | Abcam | AB290729 | Rabbit | 1:200 |

| **Primers** | **Directionality** | **Sequence** |
| --- | --- | --- |
| USP18 | Forward | CAACGTGCCCTTGTTTGTC |
| USP18 | Reverse | TGCAGTCTCTCCACCAAGTG |
| RIG-I | Forward | CACCTCAGTTGCTGATGAAGGC |
| RIG-I | Reverse | GTCAGAAGGAAGCACTTGCTACC |
| GAPDH | Forward | ACCACAGTCCATCGCATCAC |
| GAPDH | Reverse | TCCACCACCCTGTTGCTGTA |
| ISG15 | Forward | ACTCATCTTTGCCAGTACAGG |
| ISG15 | Reverse | CAGCTCTGACACACCGACATG |
| MDA5 | Forward | TCGAATGGGTATTCCACAGACG |
| MDA5 | Reverse | GGTGGCGACTGTCCTCTGAA |
| MAVS | Forward | ACCTGCACACTCTCAGGGAA |
| MAVS | Reverse | GGTCCGAGGTCCGAGGC |
| RNR1 Heavy | Bait | CGCAAATGGGCGGTAGGCGTGAGGGCTAACCATAGTGGGGT |
| RNR1 Light | Bait | CGCAAATGGGCGGTAGGCGTGCCCCTCCCCAATAAAGCTAAA |
| ND6 Heavy | Bait | CGCAAATGGGCGGTAGGCGTGGGTTGAGGTCTTGGTGAGTG |
| ND6 Light | Bait | CGCAAATGGGCGGTAGGCGTGCCCATAATCATACAAAGCCCC |
| ND5 Heavy | Bait | CGCAAATGGGCGGTAGGCGTGTTTGGGTTGAGGTGATGATG |
| ND5 Light | Bait | CGCAAATGGGCGGTAGGCGTGCATTGTCGCATCCACCTTTA |
| CO1 Heavy | Bait | CGCAAATGGGCGGTAGGCGTGTTGAGGTTGCGGTCTGTTAG |
| CO1 Light | Bait | CGCAAATGGGCGGTAGGCGTGGCCATAACCCAATACCAAACG |
| CYTB Heavy | Bait | CGCAAATGGGCGGTAGGCGTGGGATAGTAATAGGGCAAGGACG |
| CYTB Light | Bait | CGCAAATGGGCGGTAGGCGTGCAATTATACCCTAGCCAACCCC |
| RNR1 Heavy | Forward | CCCCTCCCCAATAAAGCTAAA |
| RNR1 Light | Forward | AGGGCTAAGCATAGTGGGGT |
| ND6 Heavy | Forward | TCATACTCTTTCACCCACAGC |
| ND6 Light | Forward | TGCTGTGGGTGAAAGAGTATG |
| ND5 Heavy | Forward | CTAGGCCTTCTTACGAGCC |
| ND5 Light | Forward | TAGGGAGAGCTGGGTTGTTT |
| CO1 Heavy | Forward | GCCATAACCCAATACCAAACG |
| CO1 Light | Forward | TTGAGGTTGCGGTCTGTTAG |
| CYTB Heavy | Forward | CAATTATACCCTAGCCAACCCC |
| CYTB Light | Forward | GGATAGTAATAGGGCAAGGACG |
| Universal Reverse Primer | Reverse | CGCAAATGGGCGGTAGGCGTG |
| RNR2-1 | Forward | ACCCTCACTGTCAACCCAACACAG |
| RNR2-2 | Reverse | GGCGGTGCCTCTAATACTGGTG |
| COX3-1 | Forward | CCTTTTACCACTCCAGCCTAGCC |
| COX3-2 | Reverse | CTCCTGATGCGAGTAATACGGATGT |
| ND5-1 | Forward | CTAGGCCTTCTTACGAGCCAAAACC |
| ND5-2 | Reverse | TTTGGGTTGAGGTGATGATGGAGG |

| **Chemical Reagents** | **Manufacturer** | **Catalogue Number** | **Concentration Used** |
| --- | --- | --- | --- |
| SYBRÔ Safe DNA Gel Stain | Invitrogen | S33102 |  |
| Genomic DNA Mini Kit | Bioland Scientific | GD01-01 |  |
| GeneJet RNA Purification kit | Thermofisher | Thermofisher |  |
| Kapa Sybr Fast Rox low | Roche | KK4621 |  |
| iScript SuperMix | Bio-Rad | 1708841 |  |
| DNase I, RNase free(1U/uL) | Thermofisher | EN0521 |  |
| Taq Polymerase | NEB | M0273S |  |
| dNTPs | NEB | N0447S |  |
| 10X Standard Taq Reaction Buffer | NEB | B9014S |  |
| 6X Gel Loading buffer | NEB | B7025S |  |
| NP-40 lysis buffer | ThermoFisher | J60766-AK | 1x |
| Proteinase K | ThermoFisher | EO0491 | 1 mg/mL |
| Poly-A polymerase | NEB | M0276L | 5 units |
| J2 antibody | Fisher Scientific | 10010500 | 10 ug |
| Pierce protein A/G magnetic beads | ThermoFisher | 88803 |  |
| B-Mercaptoethanol | EMD Millipore | 444203-250mL | 5% in 5x sample buffer |
| Acrylamide | Fisher Scientific | O1065-500 |  |
| Ammonium Persulfate | Sigma | A3678-100G |  |
| TEMED | Fisher Scientific | BP150-20 |  |
| Tris base | Fisher Scientific | BP152-10 |  |
| EDTA | EMD | EX0539-5 |  |
| SDS | Fisher Scientific | S529-3 |  |
| Sodium Deoxycholate | Fisher Scientific | J62288 |  |
| Triton-x 100 | Fisher Scientific | BP151-500 |  |
| Sodium Chloride | Fisher Scientific | S271-3 |  |
| Glycerol | Fisher Scientific | G33-20 |  |
| Bromophenol Blue | Fisher Scientific | B-392 |  |
| Glycine | Fisher Scientific | BP381-5 |  |
| ProLong™ Gold Antifade Mountant with DNA Stain DAPI | Invitrogen | P36935 |  |
| 16% Formaldehyde Solution (w/v), Methanol-free | Thermo Scientific | 28908 |  |
| ADAR siRNA | Ambion by Life Technologies | 4390824,  siRNA ID: s1007 |  |
| Mitotracker Red CMXROS | Invitrogen | M7512 |  |

| **Cell Lines** | **ATCC Identifier** |
| --- | --- |
| H838 | CRL-5844 |
| H1437 | CRL-5872 |
| H522 | CRL-5810 |
| A549 | CCL-185 |
| H23 | CRL-5800 |
| H441 | CVL-1561 |
| H1975 | CRL-5908 |
| H1650 | CRL-5883 |
| PC9 | Shackelford Lab |
| **Software** | **website** |
| Biorender | Biorender.com  Licensing of the figures can be found at the end of this manuscript. |
| Snapgene | Snapgene.com |
| IGV | Igv.org |
