# Supplementary material for: Characterization of Mitochondrial Double-Stranded RNA Levels in Non–Small Cell Lung Carcinoma
Source: Cancer Res Commun. 2026 Apr 7;6(4):769–82. doi: 10.1158/2767-9764.CRC-25-0656 (PMC13054796; doi:10.1158/2767-9764.CRC-25-0656)
Supplement: Supplementary Table 2 — RNA read counts analysis [file crc-25-0656_supplementary_table_2_suppst2.docx]

**Supplementary Table 2: RNA Read Counts Analysis**

Read counts using human_mito_no tRNA.gff3 file as a reference. CDS = Coding Sequence

**__**
