# Supplementary material for: Characterization of Mitochondrial Double-Stranded RNA Levels in Non–Small Cell Lung Carcinoma
Source: Cancer Res Commun. 2026 Apr 7;6(4):769–82. doi: 10.1158/2767-9764.CRC-25-0656 (PMC13054796; doi:10.1158/2767-9764.CRC-25-0656)
Supplement: Supplementary Table 3 — RNA read counts with tRNA [file crc-25-0656_supplementary_table_3_suppst3.docx]

**Supplementary Table 3: RNA Read Counts Analysis with tRNAs.**

Read counts using human_mito.gff3 file as reference. CDS = Coding Sequence
